# Optimal Placement of Unified Power Flow Controllers to Improve Dynamic Voltage Stability Using Power System Variable Based Voltage Stability Indices

**DOI:** 10.1371/journal.pone.0123802

**Published:** 2015-04-15

**Authors:** Fadi M. Albatsh, Shameem Ahmad, Saad Mekhilef, Hazlie Mokhlis, M. A. Hassan

**Affiliations:** 1 Power Electronics and Renewable Energy Research Laboratory (PEARL), Department of Electrical Engineering, University of Malaya, Kuala Lumpur, Malaysia; 2 Department of Electrical Engineering, Faculty of Engineering, University of Malaya, Kuala Lumpur, Malaysia; 3 Department of Engineering Design and Manufacture, University of Malaya, Kuala Lumpur, Malaysia; Glasgow University, UNITED KINGDOM

## Abstract

This study examines a new approach to selecting the locations of unified power flow controllers (UPFCs) in power system networks based on a dynamic analysis of voltage stability. Power system voltage stability indices (VSIs) including the line stability index (*LQP*), the voltage collapse proximity indicator (*VCPI*), and the line stability index (*L_mn_*) are employed to identify the most suitable locations in the system for UPFCs. In this study, the locations of the UPFCs are identified by dynamically varying the loads across all of the load buses to represent actual power system conditions. Simulations were conducted in a power system computer-aided design (PSCAD) software using the IEEE 14-bus and 39- bus benchmark power system models. The simulation results demonstrate the effectiveness of the proposed method. When the UPFCs are placed in the locations obtained with the new approach, the voltage stability improves. A comparison of the steady-state VSIs resulting from the UPFCs placed in the locations obtained with the new approach and with particle swarm optimization (PSO) and differential evolution (DE), which are static methods, is presented. In all cases, the UPFC locations given by the proposed approach result in better voltage stability than those obtained with the other approaches.

## Introduction

The demand for electricity has significantly increased in recent years. To meet the increasing demand, new power system networks must be constructed or existing networks must be expanded. However, developing new systems is expensive and requires a considerable amount of time. Therefore, power utilities are compelled to maximize the use of their available resources [1]. Consequently, the existing power transmission lines become more heavily loaded, and they must be operated closer to their maximum stability limits and for longer periods of time, resulting in a higher probability of voltage instability. Voltage instability is mainly associated with the inability of the power system to maintain acceptable voltages at all buses in the network [2,3]. Various control strategies, such as generation and energy transfer rescheduling, bringing standby generators online, load shedding, and volt–ampere reactive (VAR) support through series or shunt capacitors, are commonly used to improve voltage stability [4–6]. However, most of these methods require electro-mechanical controllers, which are slow and subject to wear. The Electric Power Research Institute has proposed the use of flexible alternating current transmission systems (FACTS) devices [7], which are based on self-commutated semiconductor devices. Among the various types of FACTS devices, the unified power flow controller (UPFC) is the most effective because it can be used for voltage regulation, series compensation, phase angle regulation, and control of active or reactive power separately [8]. UPFCs can solve numerous power system problems including voltage instability without the drawbacks of electromechanical devices. However, UPFCs must be placed at suitable locations in the power system network to maximize their functionality. The techniques used for selecting the locations of FACTS devices can be broadly classified into optimization-based and index-based methods [9–11].

In previous studies, multi-objective optimization techniques have been used to select the locations of FACTS devices to improve the static voltage stability of a power system network. For example, to determine the optimal location and the parameter settings of a UPFC to improve voltage stability, a differential evolution (DE) technique was used in [12–14]. Particle swarm optimization (PSO) and genetic algorithms (GA) were implemented in [15–18] and [19–21], respectively, for choosing the locations for FACTS devices, where the objectives were congestion relief and voltage stability. Non-dominated-sorting PSO (NSPSO) was used in [22,23] to find the optimal locations of thyristor controlled series capacitors (TCSCs) and static VAR compensators (SVCs) to maximize the voltage stability margin. Power system capacity was enhanced by selecting the UPFC locations using the immune GA, the immune PSO, and the non-dominated-sorting genetic algorithm II (NSGA-II) in [24,25]. However, optimization of the objectives is not guaranteed in these approaches [26]. Additionally, these studies used steady-state models of the FACTS devices, but these models cannot be used in real time to provide control actions that will prevent voltage collapse from occurring.

In many studies, index-based methods have been exploited to choose the locations for FACTS devices. Modal analysis and tangent vectors were used in [27,28] and [29], respectively, to select locations for FACTS devices to enhance system security. However, these methods cannot accurately estimate the collapse point because they behave nonlinearly near that point. The L-index was used in [30–32] to determine the bus bars where the collapse may originate and thus the locations for the FACTS devices. The L-index is suitable for constant power loads, but the results may be insufficient or conservative if some of the loads are not the constant-power type [33]. Other indices such as the line security margin index [34], the voltage security index [35], the security index [36], and the controllability index [37] were also used to choose the locations for UPFCs to improve voltage stability.

The main shortcoming of both optimization-based and index-based methods is that the locations of the FACTS devices are determined by analyzing the voltage stability in the steady state. The steady-state assumption is useful only for the planning and design stages of the power system network [38]. In actual power system networks, voltage instability occurs when the lines have become overloaded because of disturbances such as load variations, line trips, and generator outages, which are dynamic. If the locations of the FACTS devices are not chosen based on dynamic load variations, then the devices themselves can cause voltage instability.

In this study, a dynamic approach for selecting the locations of UPFCs is investigated. In this approach, the voltage stability is analyzed dynamically. Simulations were conducted in a power system computer-aided design (PSCAD) software. Power utilities mostly rely on PSCAD software because of its ability to provide fast and reliable measurements for complex power system dynamically. It can also provide a time domain simulation for transient power system networks. The information of PSCAD license used to conduct this research is presented in the appendix.

To analyze the voltage stability of a power system network, three voltage stability indices (VSIs), namely, the line stability indexes LQP and *L*_*mn*_ and the voltage collapse proximity indicator (VCPI), are used. The advantages of these indices are that they can be expressed as polynomials in terms of measurable system variables such as voltages, phase angles, and branch power flows, which can be easily collected from the network. The values of the VSIs for the transmission lines are calculated as the load, either real or reactive (PQ) or strictly reactive (Q), is increased by differing amounts at each of the buses. Proportional-plus-integral (PI) controllers are designed for both the shunt and series converters of the UPFCs. The IEEE 14-bus and 39-bus benchmark networks were chosen as examples, and these models were implemented using the components available in the power system computer-aided design (PSCAD) library. The performance of the networks using the UPFC locations obtained with the proposed method and with several heuristics approaches was compared to demonstrate the effectiveness of the proposed method.

The paper is organized as follows: section 2 explains the various VSIs and their implementation using the PSCAD software. The proposed methodology for selecting the locations of the UPFCs is explained in section 3. In section 4, a brief explanation of the dynamic model of a UPFC and its controller is presented. In section 5, the results obtained from simulations of the IEEE 14- and 39-bus test cases are discussed. Finally, section 6 provides a summary and conclusions.

### 1.1 Power System Voltage Stability Indices

**Fig 1:**
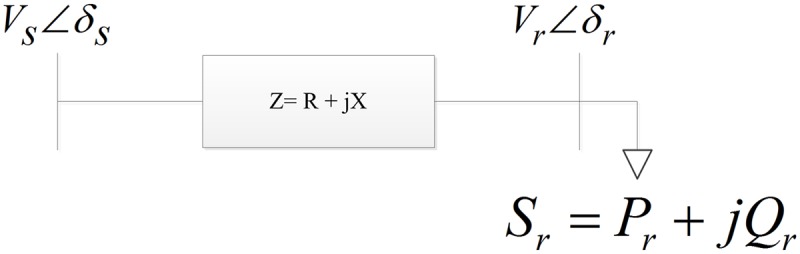
Two bus network.

Most VSIs have been formulated based on power transmission in a single line. A single line in an interconnected network is illustrated in “Fig 1”, where the suffixes *s* and *r* denote the sending and receiving ends, respectively.

Where, *V*_*s*_ and *V*_*r*_ are the sending end and receiving end voltages, respectively.

*δs* and *δr* are the phase angles at the sending and receiving buses.

*Z* is the line impedance.

*R* is the line resistance.

*X* is the line reactance.

θ is the line impedance angle.

*Qr* is the reactive power at the receiving end.

*Pr* is the active power at the receiving end.

### 1.2 L_mn_ Index

This index, which was proposed in [32], was derived for power flow through a single line, where a power system network is reduced to a single line.

From the power flow equations,

$$S_{r}\;=\;\frac{\left|V_{s}\right|\left|V_{r}\right|}{Z}\;∠(\theta -\delta_{s}+\delta_{r})-\;\frac{{\left|V_{r}\right|}^{2}}{Z}∠\theta$$ (1)

If this equation is separated in real and reactive power, then,

$$P_{r}\;=\;\frac{\left|V_{s}\right|\left|V_{r}\right|}{Z}\;\cos\;(\theta -\delta_{s}+\delta_{r})-\;\frac{{\left|V_{r}\right|}^{2}}{Z}\;\cos\theta$$ (2)

$$Q_{r}\;=\;\frac{\left|V_{s}\right|\left|V_{r}\right|}{Z}\;\sin(\theta -\delta_{s}+\delta_{r})-\frac{{\left|V_{r}\right|}^{2}}{Z}\sin\theta$$ (3)

Defining δ = *δ*_*s*_ − *δ*_*r*_ and solving eq. for *V*_*r*_, then,

$$V_{r}\;=\;\frac{V_{s}\sin(\theta -\delta )\pm {\left\{{\left[V_{s}\sin(\theta -\delta )\right]}^{2}-4ZQ_{r}\sin\theta \right\}}^{0.5}}{2\sin\theta }$$ (4)

If we substitute *Z* sin*θ* = *X* and consider the condition that the value of the square root has to be positive,

$${\left[V_{s}\sin(\theta -\delta )\right]}^{2}-4XQ_{r}\geq 0$$ (5)

Or otherwise,

$$L_{mn}\;=\;\frac{4XQ_{r}}{{\left[V_{s}\sin(\theta -\delta )\right]}^{2}}\leq 1$$ (6)

**Fig 2:**
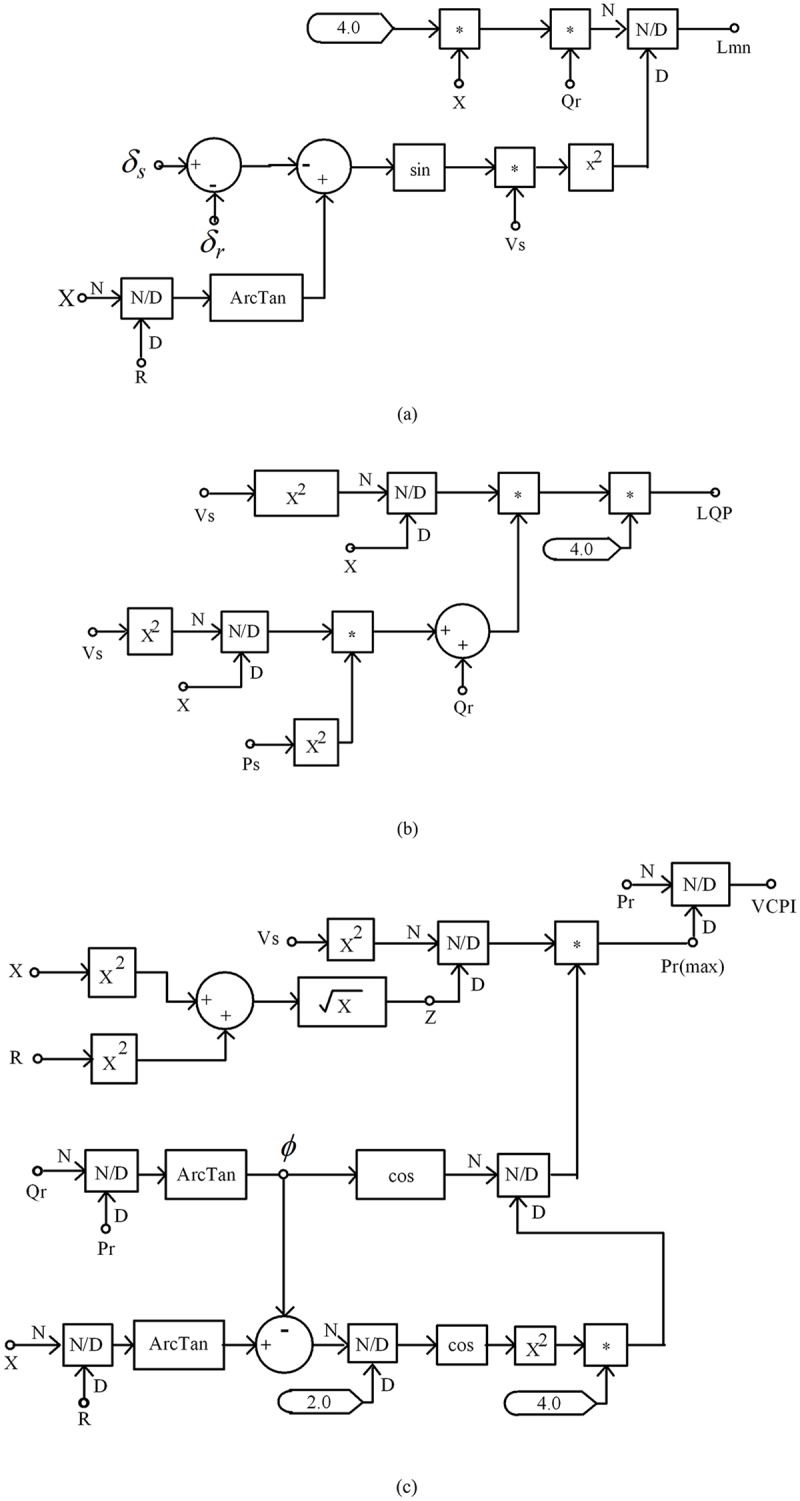
Line stability index in PSCAD (a) Lmn, (b) LQP and (c) VCPI.

This VSI is used to find the stability index for each line connection between two buses in an interconnected network. If the value of *L*_*mn*_ is less than 1, then the system is stable. The implementation of this index in the PSCAD environment is depicted in “Fig 2a”.

### 1.3 LQP Index

This index, which was defined in [36], is based on a concept similar to that of the previous index, *L*_*mn*_, and its PSCAD implementation is presented in “Fig 2b”. The index is calculated as follows:

$$LQP=4\left(\frac{V_{s}^{2}}{X}\right)\left(\frac{V_{s}^{2}}{X}P_{s}^{2}+Q_{r}\right)$$ (7)

### 1.4 Voltage Collapse Point Indicators (VCPI)

The VCPIs proposed in [34] are based on the concept of maximum power transferred through a line:

$$VCPI(P)=\frac{P_{r}}{P_{r(\max)}}$$ (8)

The numerator is the real power transferred to the receiving end and the denominator is the maximum power that can be transferred to the receiving end at a particular instant, which can be calculated as follows:

$$P_{r(\max)}=\frac{V_{s}^{2}}{Z}\frac{\cos\phi }{4\cos^{2}\left(\frac{\theta -\phi }{2}\right)}$$ (9)

Where, ∅ is the load impedance, $\phi =\tan^{-1}\frac{Q_{r}}{P_{r}}$

The implementation of this index in PSCAD is shown in “Fig 2c”.

## Methodology

**Fig 3:**
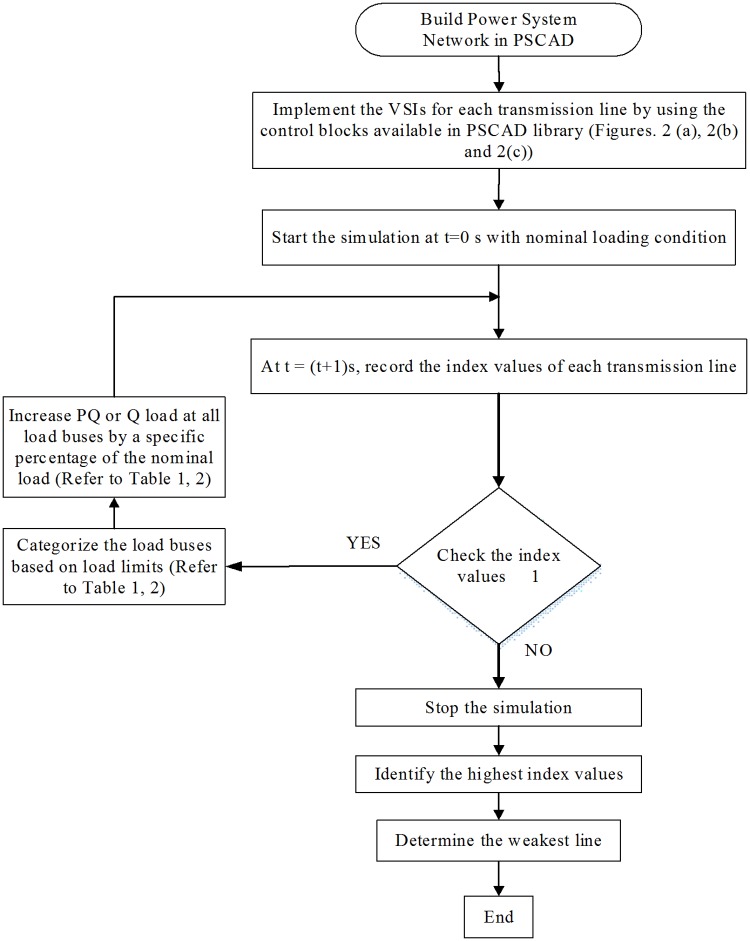
Flow chart of the proposed approach.

A flowchart of the proposed approach for selecting the locations of the UPFCs is presented in “Fig 3”. In this approach, the values of the three VSIs, LQP, L_mn_, and VCPI, are calculated for all of the transmission lines using one of two types of load variation. In the first type, PQ loads are added in all of the load buses by specific percentages (see Tables 1 and 2) of their nominal values until the value of a VSI in at least one transmission line exceeds 1.0. The second type is similar except that only the Q load rather than the PQ load is increased. All of the indices are formulated in the PSCAD environment using real-time measurements of the power system network parameters.

**Table 1:** Categories of load buses for IEEE 14 bus system under base case and load increase information for PQ load increment.

Load bus categories based on load limits	Bus	Load in Nominal condition		% of PQ load increment with respect to nominal load	Amount of load increase	
		P (MW)	Q (MVAR)		ΔP (MW)	ΔQ (MVAR)
1 (P≤20 & Q≤9)	5	7.6	1.6	30%	2.28	0.48
	6	11.2	7.5	30%	3.36	2.25
	10	9	5.8	30%	2.7	1.74
	11	3.5	1.8	30%	1.05	0.54
	12	6.1	1.6	30%	1.83	0.48
	13	13.5	5.8	30%	4.05	1.74
	14	14.9	5.0	30%	4.47	1.5
2 (P>20 & Q>9)	2	21.7	12.7	25%	5.425	3.175
	3	94.2	19	25%	23.55	4.75
	9	29.5	16.6	25%	7.375	4.15
3 (P = 47.8 &Q = 3.9)	4	47.8	3.9	20%	9.56	0.78

**Table 2:** Categories of load buses for IEEE 39 bus system under base case and load increase information for PQ load increment.

Load bus categories based on load limits	Bus	Load in Nominal condition		% of PQ load increment with respect to nominal load	Amount of load increase	
		P (MW)	Q (MVAR)		ΔP (MW)	ΔQ (MW)
1 (P≤400 & Q≤160)	3	322	2.4	20%	64.4	0.48
	7	233.8	84	20%	46.76	16.8
	12	7.5	88	20%	1.5	17.6
	15	320	153	20%	64	30.6
	16	329	32.3	20%	65.8	6.46
	18	158	30	20%	31.6	6
	21	274	115	20%	54.8	23
	23	247.5	84.6	20%	49.5	16.92
	24	308.6	92	20%	61.32	18.4
	25	224	47.2	20%	44.8	9.44
	26	139	17	20%	27.8	3.4
	27	281	75.5	20%	56.2	15.1
	28	206	27.6	20%	41.2	5.52
	29	283.5	26.9	20%	56.7	5.38
	31	9.2	4.6	20%	1.84	0.92
2 (P>400 & Q>160)	4	500	184	15%	75	27.6
	8	522	176	15%	78.3	26.4
	39	1104	250	15%	165.6	37.5
3 (P = 628 & Q = 103)	20	628	103	5%	31.4	5.15

## UPFC model and control

### 2.1 Dynamic model of a UPFC

**Fig 4:**
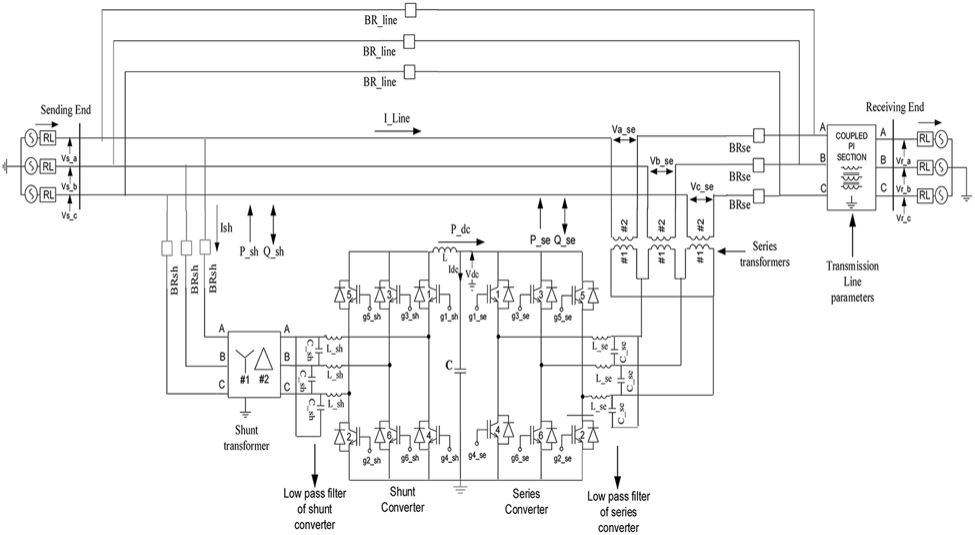
UPFC model.

A dynamic model of a UPFC as it is implemented in the PSCAD environment is shown in “Fig 4”. A UPFC consists of two voltage source converters (VSCs), namely, a shunt static synchronous compensator (STATCOM) and a series static synchronous series compensator (SSSC). These two converters are coupled via a common direct current (DC) link capacitor *C*. Two transformers, one shunt and one series, are used to connect the converters with the transmission line. The functions of these transformers are to provide isolation, to modify voltage or current levels, and to protect the DC capacitor (*C*) from being shorted during various switching operations. To prevent the flow of harmonic currents produced by switching, low-pass alternating-current filters are included in each phase. An inductor *L* is connected in parallel with the capacitor *C* to reduce the ripple in the DC voltage. Insulated-gate bipolar transistors (IGBTs) with antiparallel diodes are used as switching devices for both types of converters [39,40].

### 2.2 UPFC controllers

A UPFC controller consists of two controllers, a series controller and a shunt controller. The shunt controller draws current from the sending end of the transmission line to regulate the voltage of the DC link capacitor at its reference value. The shunt controller does this by drawing real power from the line and maintaining the transmission line voltage at its reference value by absorbing or adding reactive power in the transmission line.

A series controller controls the power flow across the line, which in turn controls the receiving-end voltage. The series controller injects a voltage in series with the line current with a controllable magnitude and angle [41].

Sinusoidal pulse–width modulation (SPWM) was used to generate the switching signals for all of the IGBTs in both the shunt and series converters. In the SPWM technique, the reference signals are compared with the carrier (triangle) signal, which has a switching frequency of 3.5 KHz, to generate the switching signals for the converter switches [42].

## Results and Discussion

**Fig 5:**
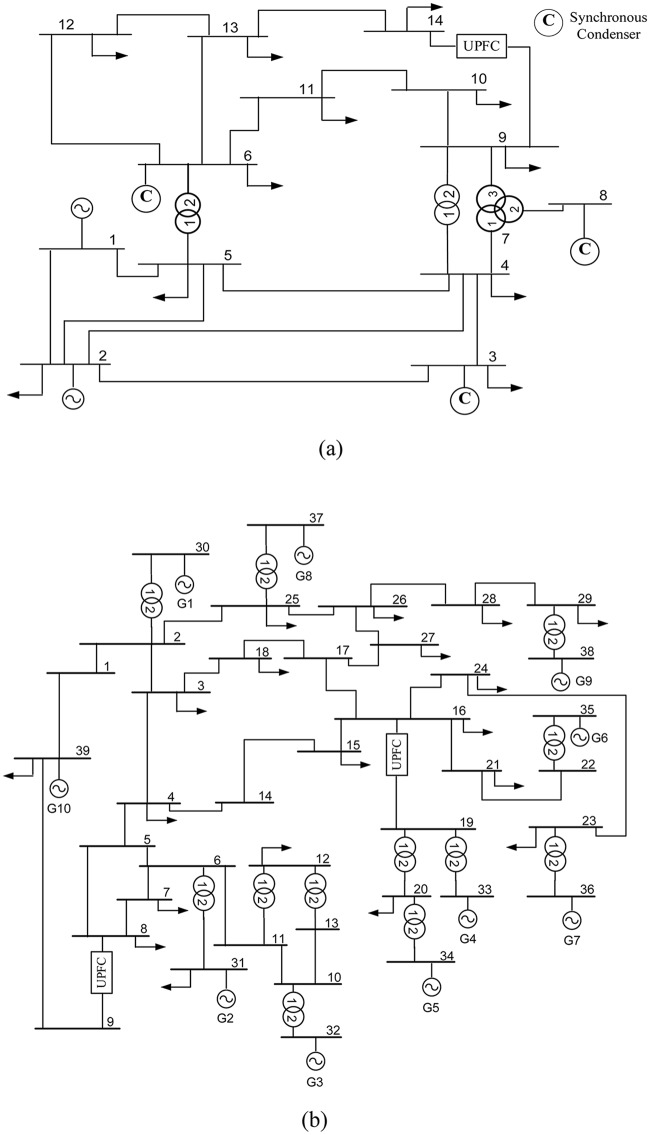
IEEE bus system network with UPFC in the location identified varying PQ load (a) IEEE 14 bus system and (b) IEEE 39 bus system.

In this section, the results obtained from simulations of the IEEE 14-bus and 39-bus systems, the test cases in this study, are presented. The power flow and dynamic data of the IEEE 14 bus system and IEEE 39 bus system networks can be found in [43].The benchmark systems were implemented in the PSCAD environment using the transient components available in the PSCAD library. Single-line diagrams of the two networks, including the UPFCs, are shown in “Fig 5a and 5b”.

Two sets of simulations were conducted. In the first set, the locations of the UPFCs were selected using the three VSIs (LQP, VCPI, and L_mn_) to analyze the voltage stability of the system under dynamic conditions. In the second set of simulations, the UPFCs were inserted at the locations obtained in the first step and the effect on voltage stability was evaluated.

Because voltage instability can be caused by variations in either type of load (PQ or Q), both types were used to choose the locations of the UPFCs; i.e., the locations of the UPFCs were chosen first by increasing both the P and Q loads at all of the load buses and then only the Q loads.

### 3.1 UPFC location selection

This section presents the methodology for selecting the locations of the UPFCs in the IEEE 14- and 39-bus systems using either PQ or Q load variations.

#### 3.1.1 Case 1: PQ load variations in IEEE 14-bus system

For this case, the PQ load was increased by differing amounts across each of the load buses simultaneously. The reason for using different amounts in the PQ load is that in practical power systems, the load variations are not identical in all of the buses.

**Fig 6:**
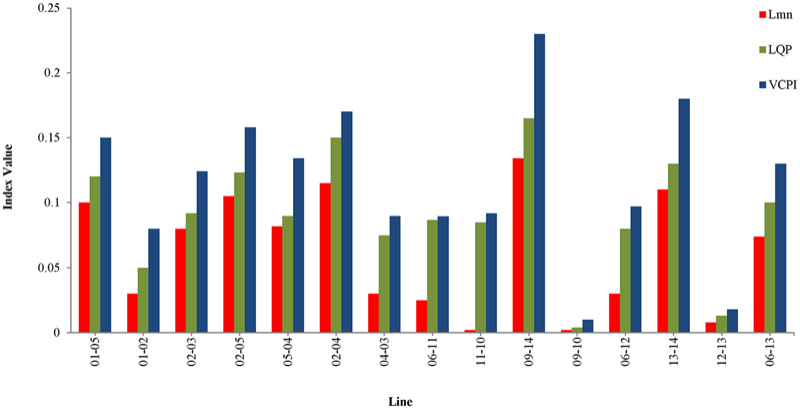
Index values of all the transmission lines for IEEE 14 bus system at nominal loading condition.

The simulation begins at t = 0 s with nominal load conditions in the network. After t = 1 s, the values of the stability indices for all of the transmissions lines are recorded. Here, a transmission line is represented by the word ‘line’ followed by the numbers of the buses across which the transmission line is connected. For example, the transmission line connected across bus 1 and bus 5 is denoted by ‘line 1–5’. It can be observed in “Fig 6” that at t = 1 s, none of the indices exceed 1.0; the highest value, 0.236, occurs in line 9–14.

Three bus categories are defined based on the nominal P and Q load limits, which are shown in Table 1. The load is incremented by different percentages of the nominal load in each category. The percentage increments in the P and Q loads in categories 1, 2, and 3 are 30%, 25%, and 20%, respectively.

**Fig 7:**
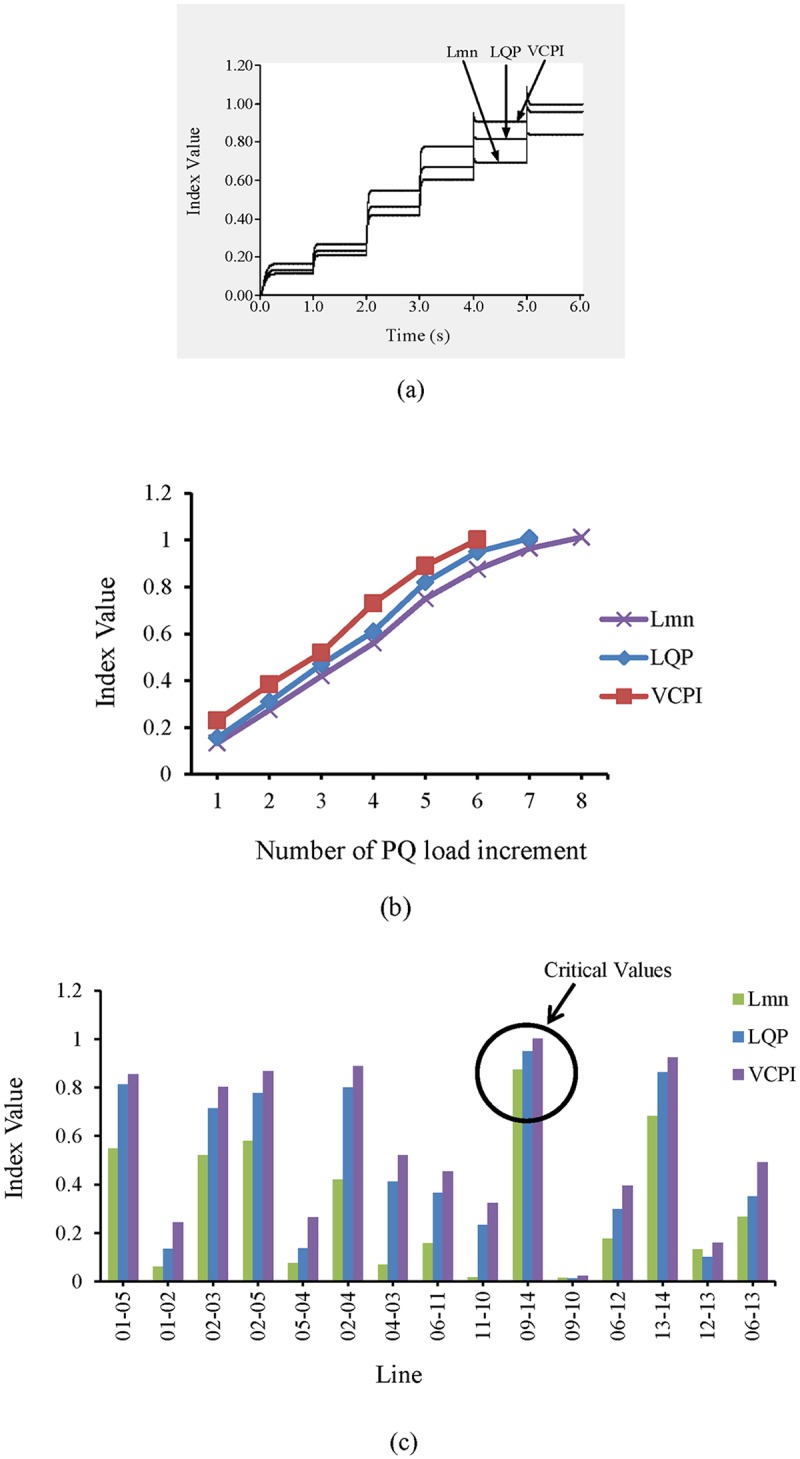
Index values for different VSIs (a) for line 9–14 of IEEE 14 bus system with respect to time (b) for line 9–14 of IEEE 14 bus system with respect to number of PQ load increment and (c) for all transmission lines of IEEE 14 bus system for maximum PQ load increment.

As shown in “Fig 7a”, line 9–14 becomes unstable when the load is increased the fifth time. In other words, the values of the VSIs are all less than 1 until t = 5 s. After t = 5 s, the load has increased by 150%, 125%, and 100% of the nominal load, and the value of the VCPI index for line 9–14 exceeds 1.0. However, for the same increase in load, the other VSIs, LQP and L_mn_, for line 9–14 for do not indicate voltage instability. Therefore, a higher load is required for the LQP index to indicate instability in line 9–14 than that required for the VCPI index. The load was increased until the L_mn_ index indicated instability in line 9–14. The values of all of the VSIs for line 9–14 for each of the load increases are depicted in “Fig 7b”. The VSI values for all of the transmission lines after the final load increase are presented in “Fig 7c”. Because the VSIs indicate that instability occurs first in line 9–14, this line was selected as the location for the UPFC.

#### 3.1.2 Case 1: PQ load variations in IEEE 39-bus system

**Fig 8:**
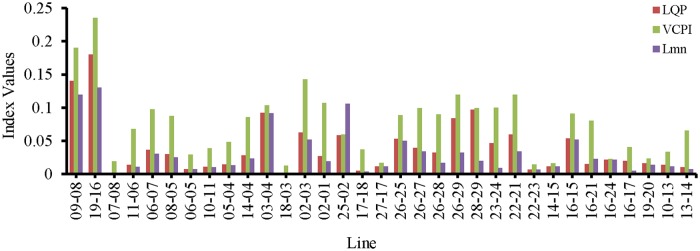
Index values of all the transmission lines of IEEE 39 bus system for nominal load.

The simulation begins at t = 0 s with nominal loading conditions on the network. At t = 1 s, the values of the VSIs for all of the lines are computed. “Fig 8” shows that none of the VSIs exceed 1.0 for any of the transmission lines. Both lines 19–16 and 9–8 have the highest VSI values, which are less than 0.25.

**Fig 9:**
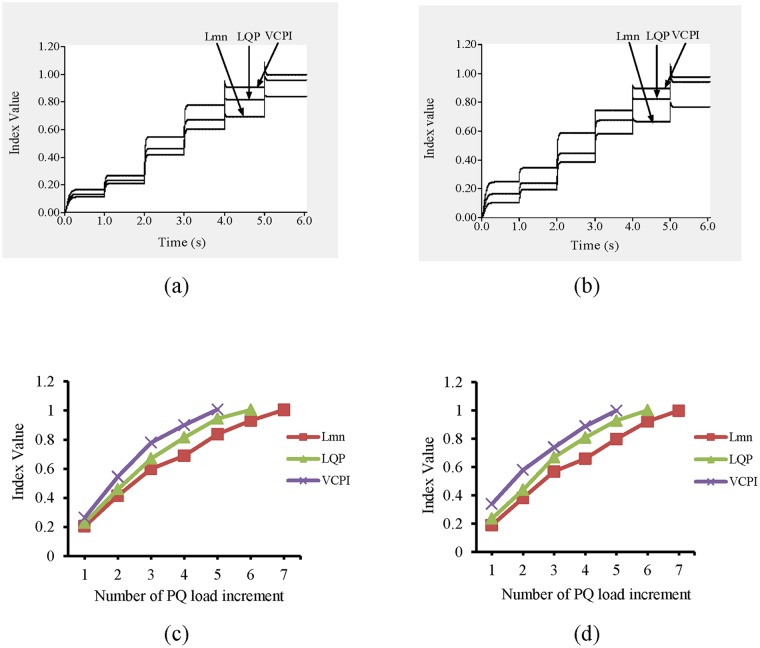
Index values of (a) line 9–8 for different VSIs with respect to time, (b) line 19–16 for different VSIs with respect to time, (c) line 9–8 for different VSIs with respect to number of PQ load increment and (d) line 19–16 for different VSIs with respect to number of PQ load increment.

In “Fig 9a and 9b”, the values of the VSIs for lines 9–8 and 19–16 with respect to time are presented. The load buses are divided into three categories based on the nominal P and Q load limits, which are shown in Table 2, and the P and Q loads on each bus are increased by different increments depending on the category. From Table 2, it can be observed that the percentage increases in the P and Q loads in categories 1, 2, and 3 are 20%, 15%, and 10%, respectively.

**Fig 10:**
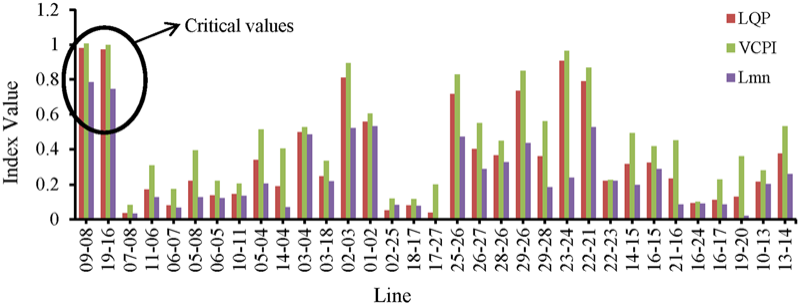
Index values of all transmission lines of IEEE 39 bus system for maximum PQ load increment.

After t = 1 s, the first increase in the load occurs, and at t = 2 s, the values of the VSIs are computed again. Again, none of the VSIs exceed 1.0. At each second, the load increases and the values of the VSIs are computed. Until t = 5 s, the values of the VSIs for all of the lines remain less than 1.0. After t = 5 s, when the load increases to 100%, 75% and 50% of the nominal load, the values of the VCPI for lines 9–8 and 19–16 exceed 1. However, for the same load the values of the LQP and L_mn_ indices for these lines remain less than 1.0. Higher loads are required to reach instability according to these two indices, but the required load is higher for the L_mn_ index than for the LQP index. The values of all of the VSIs for lines 9–8 and 19–16 with respect to the load increases are shown in “Fig 9c and 9d”, respectively. The values of the VSIs for all of the lines after the final load increase are presented in “Fig 10”. Because the VSIs exceeded 1.0 first in lines 9–8 and 19–16, these lines were selected as the locations for the UPFCs.

#### 3.1.3 Case 2: Q load variation in IEEE 14-bus system

In this case study, the locations of the UPFCs in the IEEE 14- and 39-bus systems are selected using only Q load increases over all of the buses.

In this section, the UPFC location is identified based on only Q load variations. It can be observed from “Fig 6” that under nominal loading conditions, no transmission line exhibits instability.

The load buses are divided into three categories based on the Q load limits, which are presented in Table 3. At one-second intervals, the loads across the load buses in categories 1, 2, and 3 are increased by 35%, 25%, and 20% of the nominal loads, respectively.

**Table 3:** Categories of load buses for IEEE 14 bus system under base case and load increase information for Q load increment.

Categories based on load limits	Bus	Load in Nominal condition Q (MVAR)	% of Q load increment with respect to nominal load	Amount of load increase ΔQ (MVAR)
1 (0≤Q≤9)	4	3.9	35%	1.635
	5	1.6	35%	0.56
	6	7.5	35%	2.625
	10	5.8	35%	2.03
	11	1.8	35%	0.63
	12	1.6	35%	0.56
	13	5.8	35%	2.03
	14	5.0	35%	1.75
2 (20<Q≤18)	2	12.7	25%	3.175
	9	16.6	25%	4.15
3 (Q>18)	3	19	20%	3.8

**Fig 11:**
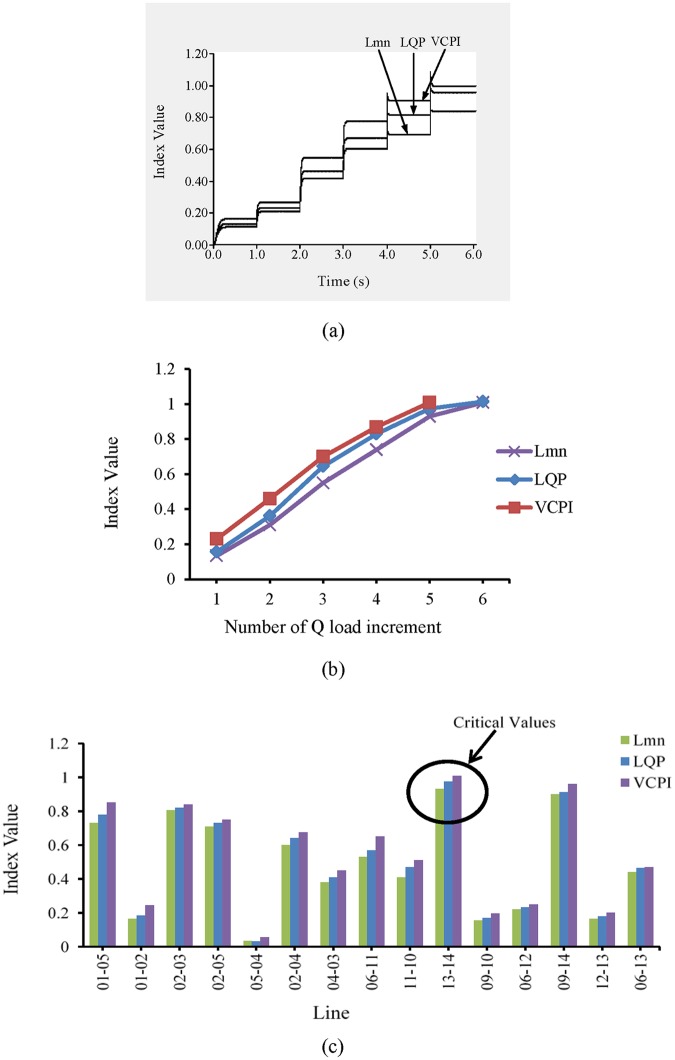
Index values for different VSIs (a) for line 13–14 of IEEE 14 bus system with respect to time, (b) for line 13–14 of IEEE 14 bus system with respect to number of Q load increment and (c) for all transmission lines of IEEE 14 bus system for maximum Q load increment.

After t = 5 s, the value of the VCPI index for line 13–14 exceeds 1.0, as shown in “Fig 11a”. At that instant, the load increases across the buses in categories 1, 2, and 3 are 140%, 100%, and 80%, respectively, of the nominal load. The values of the other two indices, L_mn_ and LQP, for this line remain less than 1.0. When the load increases again, both the L_mn_ and LQP indices indicate instability in line 13–14. The values of the VSIs for line 13–14 for Q load increases are presented in “Fig 11b”. “Fig 11c” shows the values of the VSIs for all of the lines after the final Q load increment.

#### 3.1.4 Case 2: Q load variation in IEEE 39-bus system

**Fig 12:**
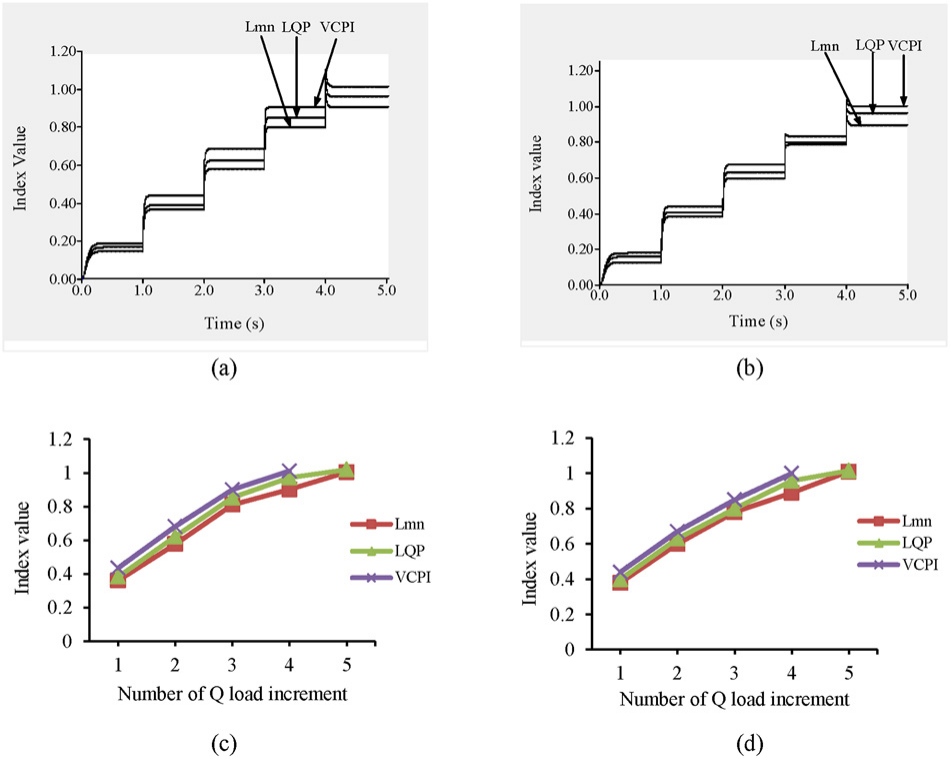
Index values for different VSIs (a) for line 9–8 with Q load increment, (b) for line 15–16 with Q load increment, (c) for line 9–8 with respect to number of Q load increment and (d) for line 15–16 with respect to number of Q load increment.

**Fig 13:**
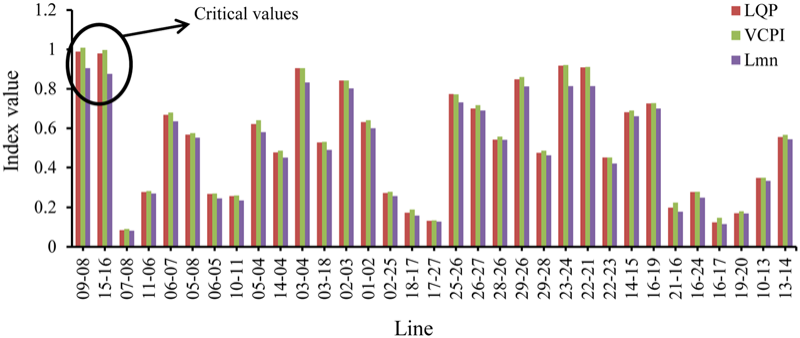
Index values of all transmission lines of IEEE 39 bus system for maximum Q load increment.

It can be observed from “Fig 13” that at t = 1 s, when the load on the system is nominal, none of the lines exhibits instability. The load buses are divided into three categories based on the Q load limits. The categories of the load buses are presented in Table 4. At one-second intervals, the loads on the buses in categories 1, 2, and 3 increase by 30%, 25%, and 20% of the nominal loads, respectively. The values of the VCPI index for two lines, 9–8 and 15–16, exceed 1.0 at t = 5 s. The VCPI indices for lines 9–8 and 15–16 are presented in “Fig 12a and 12b”, respectively. At that moment, the load increases across all the buses in categories 1, 2, and 3 are 120%, 100%, and 80%, respectively, of the nominal load. For the same load, the values of the remaining VSIs (L_mn_ and LQP) for the lines are less than 1.0, indicating that the system is stable. When the load increases again, the values of both the L_mn_ and LQP indices for lines 9–8 and 15–16 exceed 1.0. The values of these two indices for lines 9–8 and 15–16 with Q load variations are presented in “Fig 12c and 12d”.

**Table 4:** Categories of load buses for IEEE 39 bus system under base case and load increase information for Q load increment.

Categories based on load limits	Bus	Load in Nominal condition Q (MVAR)	% of Q load increment with respect to nominal load	Amount of load increase ΔQ (MW)
1 (0≤Q≤70)	3	2.4	30%	0.72
	16	32.3	30%	9.69
	18	30	30%	9
	25	47.2	30%	14.16
	26	17	30%	5.1
	28	27.6	30%	8.28
	29	26.9	30%	8.07
	31	4.6	30%	1.38
2 (70<Q≤160)	7	84	25%	21
	12	88	25%	22
	15	153	25%	38.25
	20	103	25%	25.75
	21	115	25%	28.75
	23	84.6	25%	21.15
	24	92	25%	23
	27	75.5	25%	18.875
3 (Q>160)	4	184	20%	36.8
	8	176	20%	35
	39	250	20%	50

“Fig 13” shows the values of the VSIs for all of the lines after final Q load increase. In this case, the locations chosen for the UPFCs are lines 9–8 and 15–16.

### 3.2 Voltage stability improvement with UPFCs

In this section, the IEEE 14-bus and 39-bus systems are simulated again with the UPFCs inserted at the locations (transmission lines) identified in the previous section. The PQ and Q loads are varied, and the voltage stability is evaluated again.

#### 3.2.1 Effect of UPFCs on voltage stability with PQ load variations in IEEE 14-bus system

The effect of the UPFCs on voltage stability in the two power system networks with variations in the PQ load is examined in this section.

**Fig 14:**
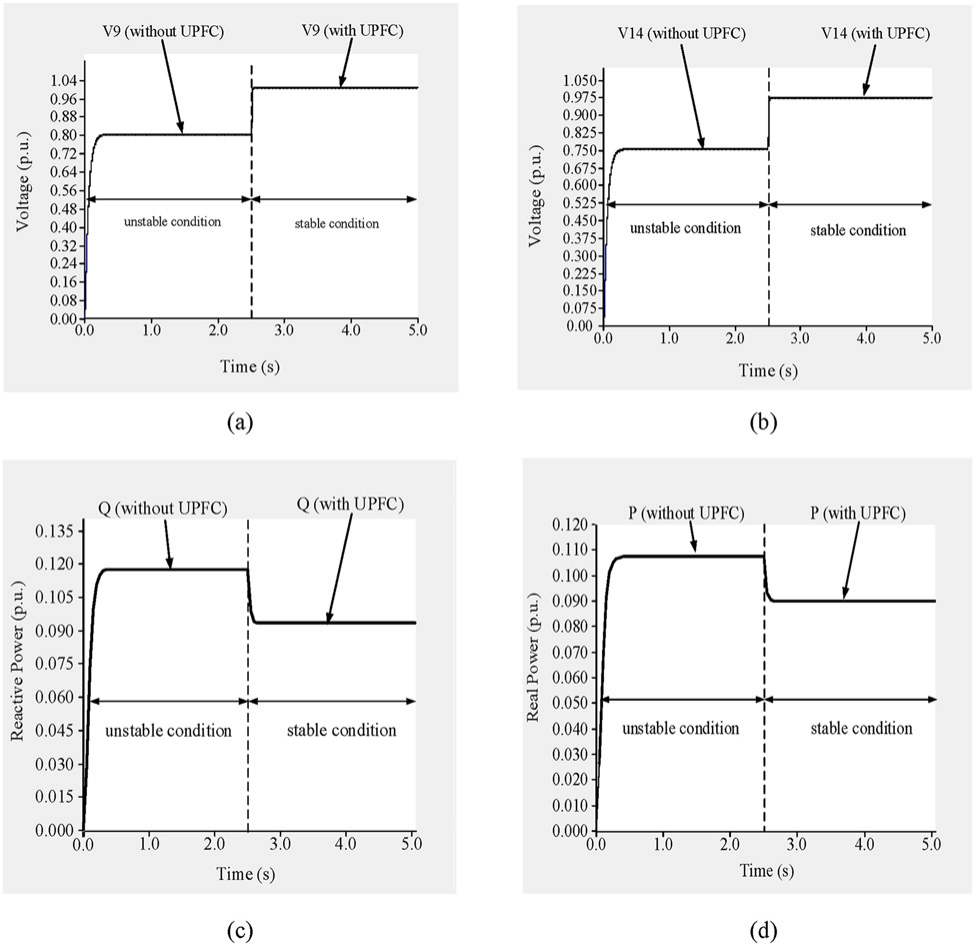
(a)Voltage across bus 9, (b) voltage across bus 14, (c) reactive power flow through line 9–14 and (d) real power flow through line 9–14 of IEEE 14 bus system.

To improve the voltage stability, a UPFC was placed across line 9–14. Simulation results are shown in “Fig 14”. From 0 to 2.5 s, before the UPFC is connected, the network is unstable, and during that period the voltages across buses 9 and 14 are 0.89 p.u. and 0.728 p.u., respectively, as shown in “Fig 14a and 14b”.

From “Fig 14c and 14d”, the reactive and real power flows at that time are 0.1189 p.u. and 0.1055 p.u., respectively. The reference levels of reactive and real power through line 9–14 are 0.093 p.u. and 0.090 p.u., respectively, and the UPFC bus voltage reference is 0.99 p.u.

At 2.5 s, the UPFC is connected, after which the bus voltages increase and the voltage stability improves. From 2.5 to 5.0 s, the voltages across buses 9 and 14 are 1.012 p.u. and 0.9875 p.u., respectively, as shown in “Fig 14a and 14b”. In addition, the reactive and real power flows are 0.094 p.u. and 0.092 p.u., respectively, as shown in “Fig 14c and 14d”.

**Fig 15:**
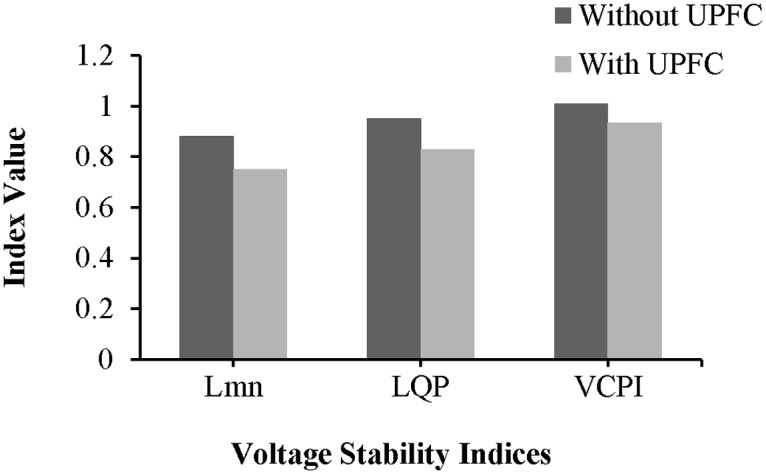
Index values of line 9–14 of IEEE 14 bus system.

Without the UPFC and in the unstable condition, the values of the L_mn_, LQP, and VCPI indices for line 9–14 are 0.8745, 0.935, and 1.008, respectively, as shown in “Fig 15”. The values of these indices decrease after the UPFC is connected to line 9–14: the values of the L_mn_, LQP, and VCPI indices are 0.8045, 0.866, and 0.934, respectively.

**Fig 16:**
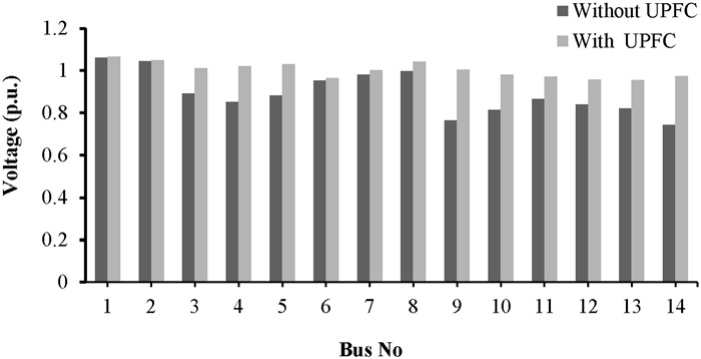
Voltage profile across all the buses of IEEE-14 bus network with UPFC placement in the line 9–14.

The placement of the UPFC across line 9–14 improved the bus voltages, which are plotted in “Fig 16”.

#### 3.2.2 Effect of UPFCs on voltage stability with PQ load variations in IEEE 39-bus system

**Fig 17:**
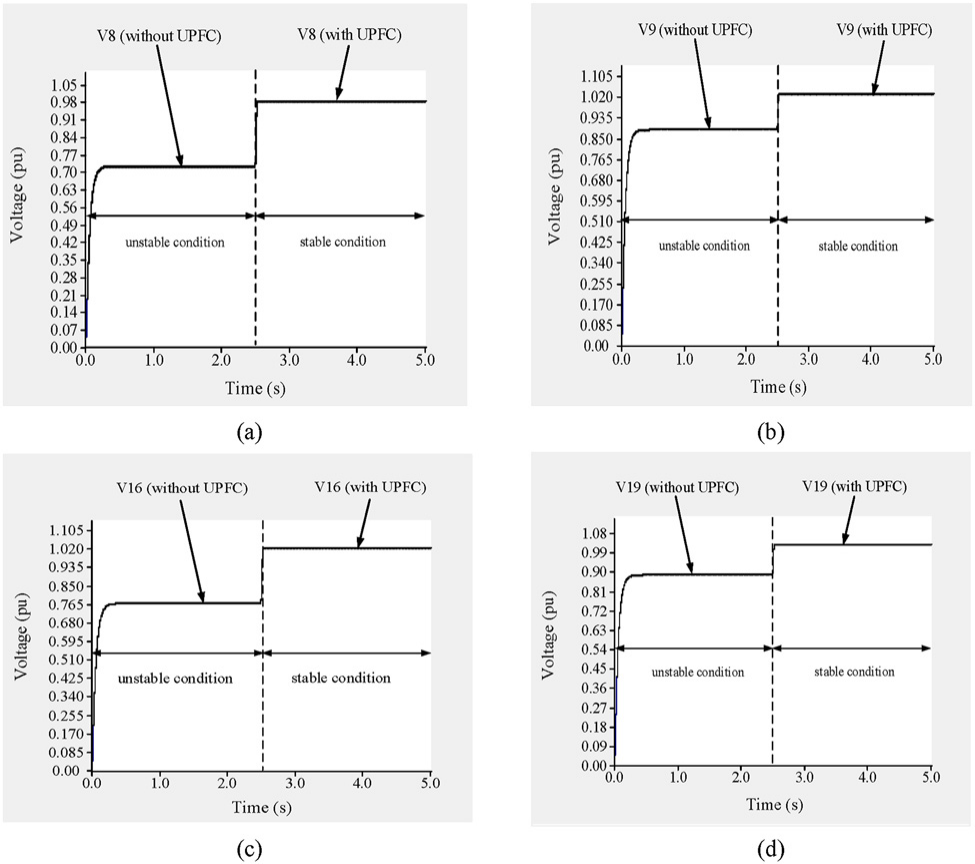
(a) Voltage across bus 8, (b) Voltage across across bus 9, (c) Voltage across bus 16 and (d) Voltage across bus 19 of IEEE 39 bus system.

**Fig 18:**
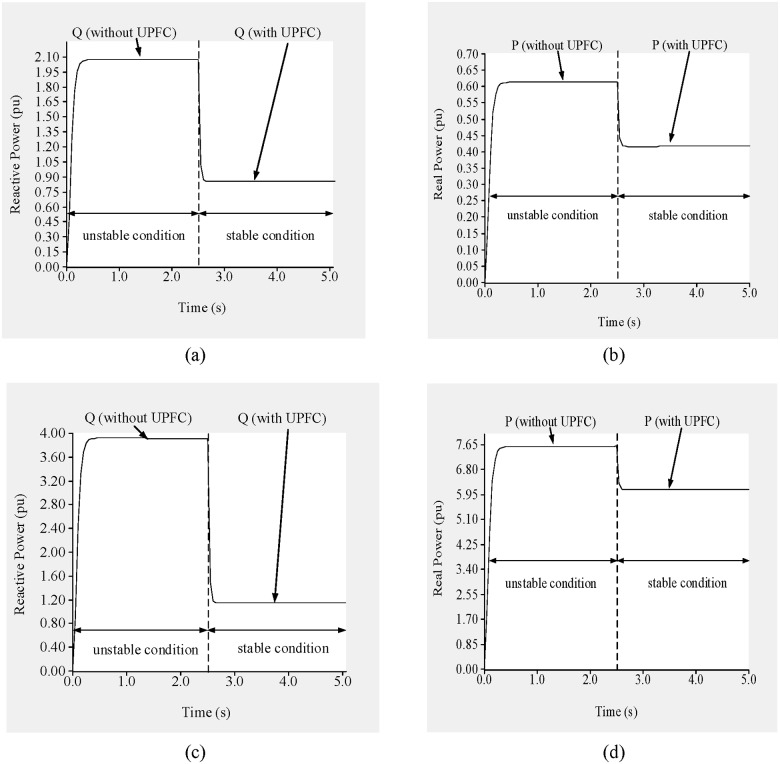
(a) reactive power flow through line 9–8, (b) real power flow through line 9–8, (c) reactive power flow through line 19–16 and (d) real power flow through line 19–16 of IEEE 39 bus system.

In this example, UPFCs are placed across lines 9–8 and 19–16. The simulation results of the bus voltages and the power across lines 9–8 and 19–16 are shown in “Figs 17 and 18”. From 0 to 2.5 s, the UPFCs are not connected and the system is in an unstable condition. The voltages across buses 8, 9, 16, and 19 are 0.7235 p.u., 0.8735 p.u., 0.78 p.u., and 0.8927 p.u., respectively, as shown in “Fig 17”.

In addition, as shown in “Fig 18a and 18b”, the flows of reactive and real power through line 9–8 are 2.05 p.u. and 0.602 p.u., respectively, and the reactive and real power flows through line 19–16 are 3.77 p.u. and 7.65 p.u., respectively, as shown in “Fig 18c and 18d”.

Based on the previous results, the reference values of the reactive and real power flows across line 9–8 are set to 0.95 p.u. and 0.45 p.u., respectively, and the sending-end voltage is set to 0.99 p.u. to bring the system to a stable condition. Similarly, for line 19–16 the reference reactive and real power levels are set to 1.40 p.u. and 6.0 p.u., respectively, and the UPFC bus voltage is restricted to 1.01 p.u.

At 2.5 s, the UPFCs are connected to the lines. As shown in “Fig 17a and 17b”, the voltages across buses 8 and 9 are 0.986 p.u. and 1.016 p.u., respectively. In addition, the voltage across buses 16 and 19, as shown in “Fig 17c and 17d”, increase to 1.002 p.u. and 1.03 p.u., respectively. From 2.5 to 5.0 s, the flows of reactive and real power across lines 9–8 and 19–16 approach their nominal values, as can be observed in “Fig 18”. From “Fig 18a and 18b”, it can be observed that the flows of both reactive and real power across line 9–8 reach 0.935 p.u. and 0.441 p.u., respectively. For line 19–16, the flows of reactive and real power, as shown in “Fig 18c and 18d”, are 1.38 p.u. and 5.875 p.u., respectively.

**Fig 19:**
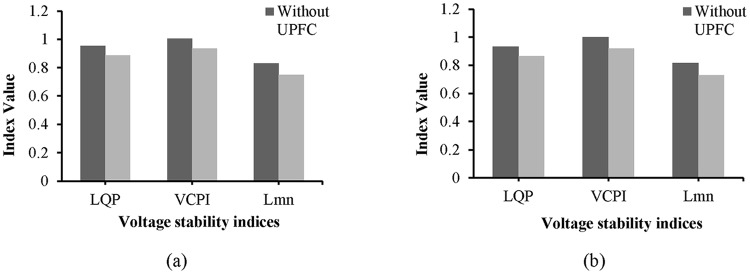
Index values of (a) line 9–8 and (b) line 19–16 of IEEE 39 bus system.

From “Fig 19a”, the values of the L_mn_, LQP, and VCPI indices for line 9–8 before the UPFCs are connected are 0.8378, 0.943, and 1.006, respectively, and after connecting the UPFCs, the values are 0.7532, 0.886, and 0.934. “Fig 19b” shows the values of the VSIs for line 19–16, where it can be observed that the L_mn_ index decreases from 0.828 to 0.753, the LQP index decreases from 0.934 to 0.8623, and the VCPI decreases from 1.001 to 0.9215 with the UPFCs connected. The decrease in the values of the VSIs for lines 9–8 and 19–16 indicate that the voltage stability of the system has improved.

**Fig 20:**
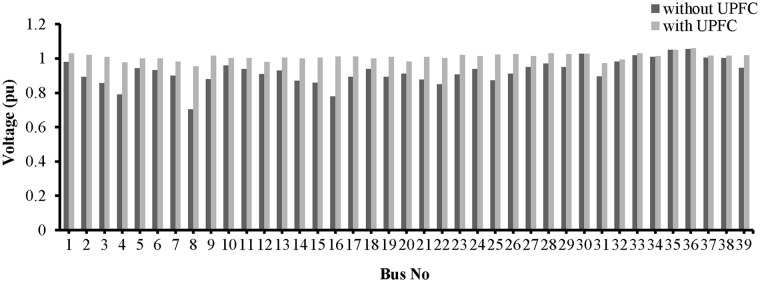
Voltage profile across all the buses of IEEE-39 bus network with UPFCs placement in lines 9–8 and 19–16.

The voltage profiles for all of the buses are shown in “Fig 20”. The placement of the UPFCs across lines 19–16 and 9–8 has improved the voltages across all of the buses.

#### 3.2.3 Effect of the UPFCs on voltage stability with Q load variations in IEEE 14-bus system

The effect of UPFCs on voltage stability in the IEEE 14- and 39-bus networks with Q load variations is examined in this section.

**Fig 21:**
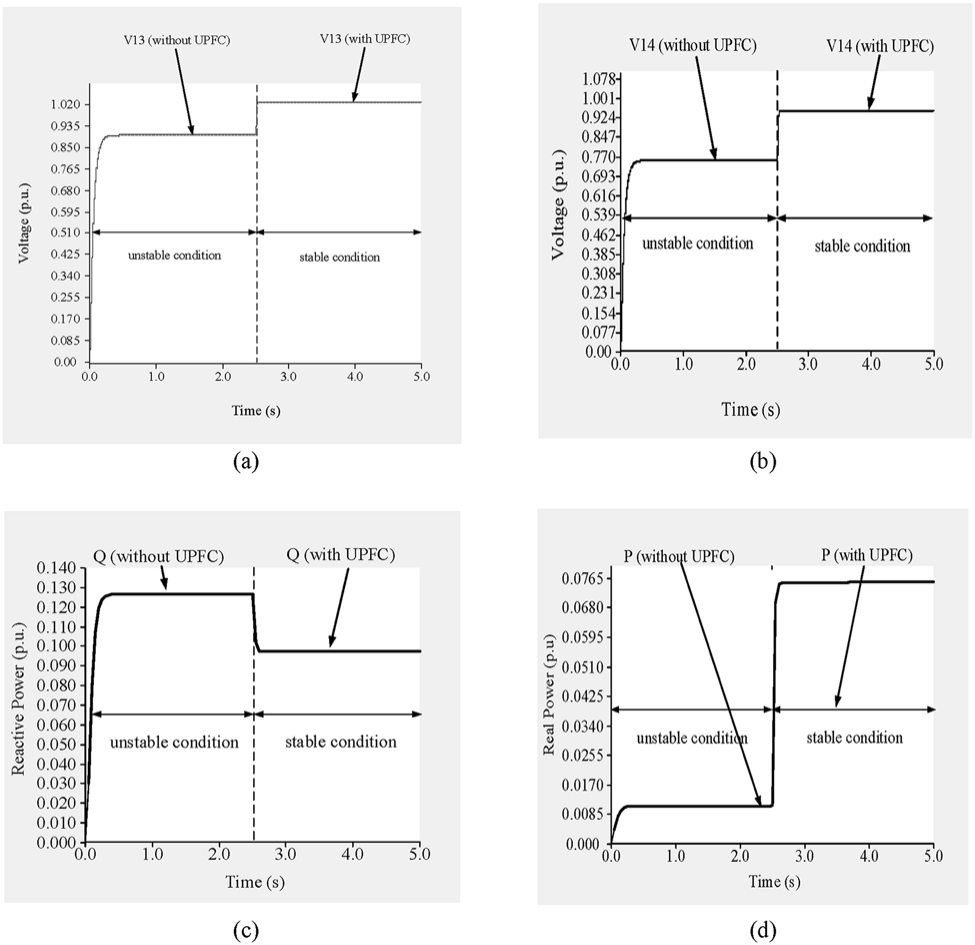
(a) Voltage across bus 13, (b) voltage across bus 14, (c) reactive power flow through line 13–14 and (d) real power flow through line 13–14 of IEEE 14 bus system.

The simulation begins without the UPFC connected for the first 2.5 s, and the network is in an unstable condition. During this period, the voltages across buses 13 and 14 are 0.89 p.u. and 0.732 p.u., respectively, as shown in “Fig 21a and 21b”.

“Fig 21c and 21d” show the reactive and real power flows, respectively, in line 13–14 for the stable and unstable conditions. The reference reactive power is set to 0.1 p.u. for a measured reactive power of 0.128 p.u. Similarly, for a measured real power of 0.0095 p.u. the reference real power is set to 0.077 p.u. The reference UPFC bus voltage is set to 0.95 p.u. At 2.5 s, the UPFC is connected to the line, which brings the system to the stable condition. From “Fig 21a and 21b”, it can be observed that in the stable condition and with the UPFC connected across line 13–14, the voltages across buses 13 and 14 improve by 13.48% (1.01 p.u.) and 27.9% (0.942 p.u.), respectively, compared with the unstable condition. In the stable condition, the reactive and real power flows are 0.1033 p.u. and 0.0765 p.u., respectively, as shown in “Fig 21c and 21d”.

**Fig 22:**
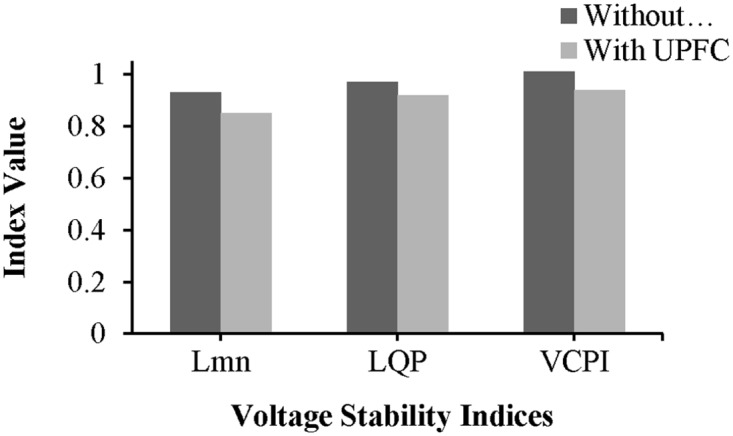
Index values of line 13–14 of IEEE 14 bus system.

The values of the VSIs for line 13–14 are presented in “Fig 22”. As shown in “Fig 22”, without the UPFC and with the network in the unstable condition, the values of the L_mn_, LQP, and VCPI indices for line 9–14 are 0.92, 0.953, and 1.005, respectively. The values of the L_mn_, LQP, and VCPI indices for line 13–14 decrease by 6.03% (0.8645), 7.13% (0.885) and 6.65% (0.945), respectively, after the UPFC is connected across the line.

**Fig 23:**
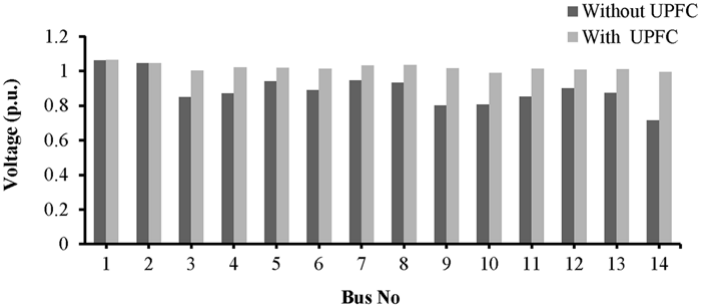
Voltage profile across all the buses of IEEE-14 bus network with UPFC placement in the line 13–14.

From “Fig 23”, it can be observed that the voltage profiles of the buses in the network improve with the UPFC in line 13–14.

### 3.2.4 Effect of the UPFCs on voltage stability with Q load variations in IEEE 39-bus system

**Fig 24:**
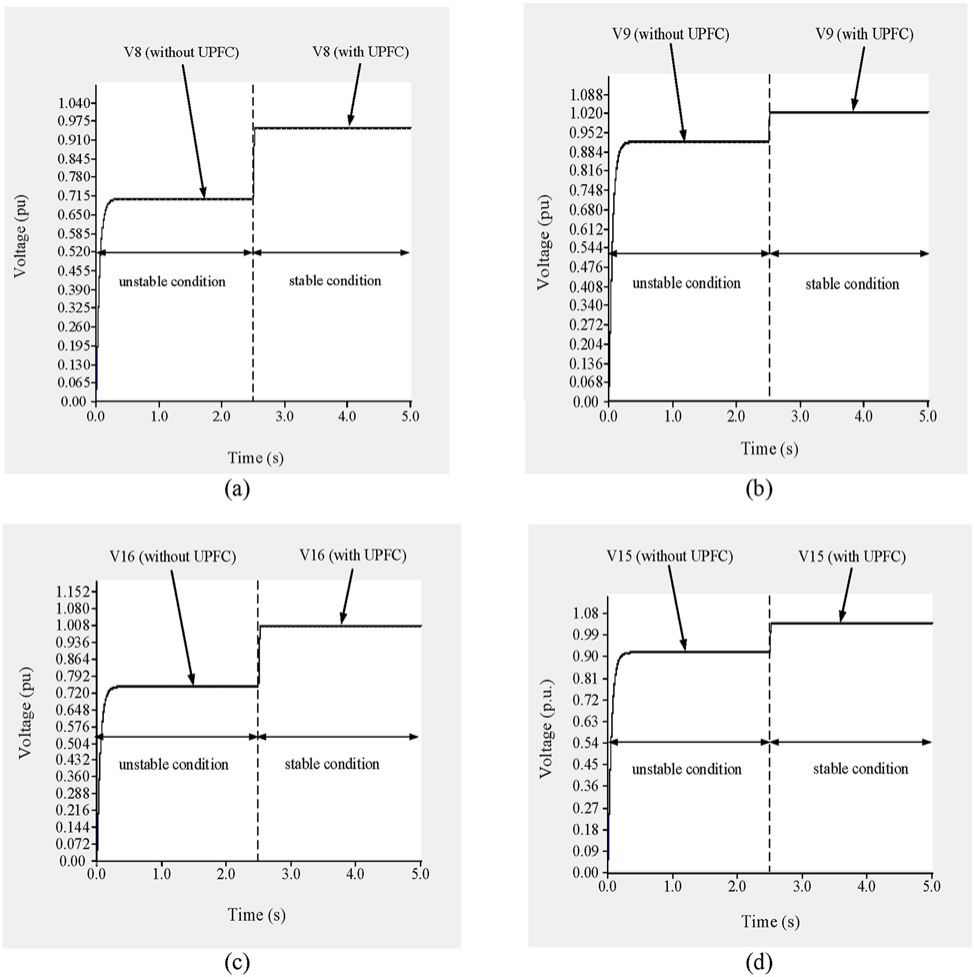
(a) Voltage across bus 8, (b) Voltage across bus 9, (c) Voltage across bus 16 and (d) Voltage across bus 15 of IEEE 39 bus system.

To improve the voltage stability, UPFCs are placed across lines 9–8 and 15–16. From 0 to 2.5 s, the system is in an unstable condition and the voltages across buses 8, 9, 16, and 15 are 0.715 p.u., 0.912 p.u., 0.759 p.u., and 0.925 p.u., respectively, as shown in “Fig 24”.

**Fig 25:**
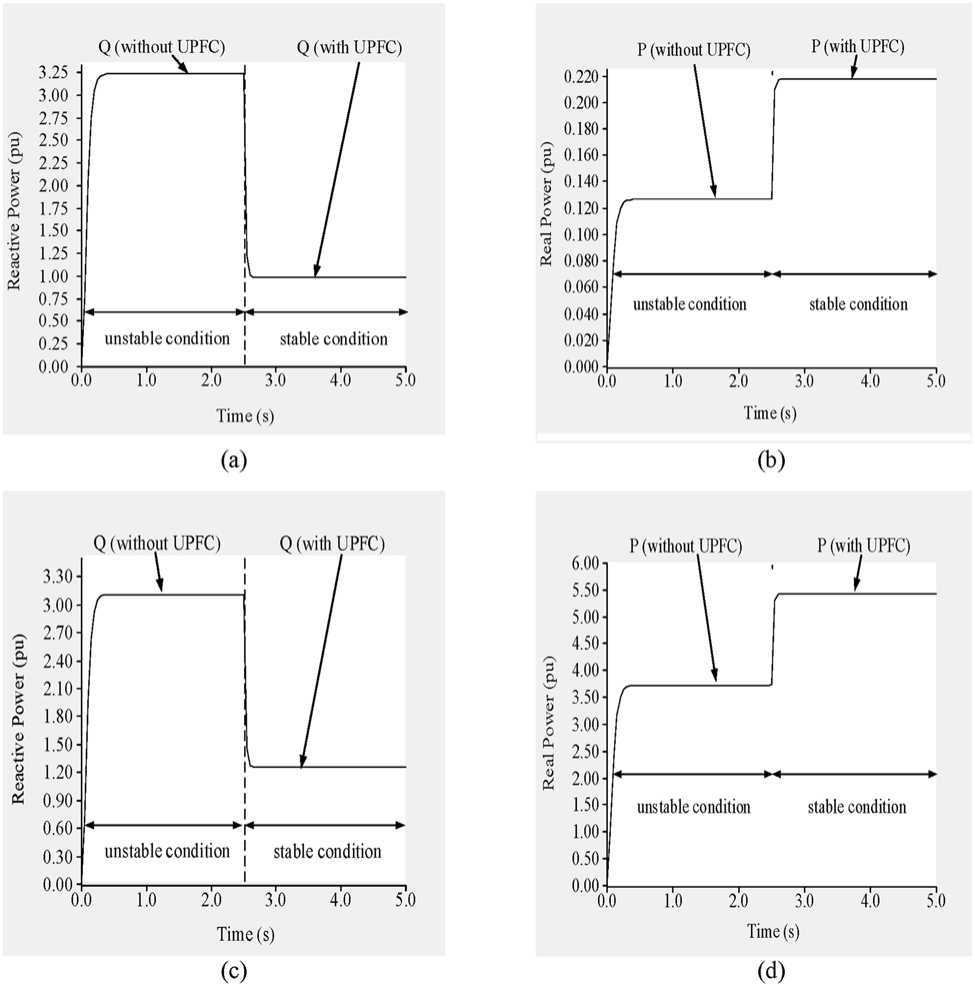
(a) reactive power flow through line 9–8, (b) real power flow through line 9–8, (c) reactive power flow through line 15–16 and (d) real power flow through line 15–16 of IEEE 39 bus system.

In addition, the reactive and real power flows through line 9–8 are 3.24 p.u. and 0.13 p.u., respectively “Fig 25a and 25b”, and 3.40 p.u. and 3.10 p.u., respectively, through line 15–16 “Fig 25c and 25d”.

To ensure the stability of the system, the reference reactive and real power levels are set to 1.15 p.u. and 0.22 p.u., respectively, for line 9–8 and 1.35 p.u. and 5.50 p.u., respectively, for line 15–16. The reference UPFC bus voltages are set to 0.97 p.u., 1.011 p.u., 1.01 p.u., and 1.02 p.u. for buses 8, 9, 16, and 15, respectively. At 2.5 s, the UPFCs are connected to the lines. From “Fig 24”, it can be observed that the voltages across buses 8, 9, 16, and 15 improve by 0.251 p.u., 0.098 p.u., 0.24 p.u. and 0.095 p.u. and reached to (0.966 p.u.), (1.01 p.u.), (1.009 p.u.) and (1.02 p.u.), respectively. As can be observed from “Fig 25a and 25c”, the reactive power flows through lines 9–8 and 15–16 decrease by 2.05 p.u. and 1.65 p.u., and reached to (1.175 p.u.) and (1.41 p.u.) respectively. While the real power flows improve by 0.09 p.u. and 2.3 p.u., and reached to (0.212 p.u.) and (5.41 p.u.) respectively, as shown in “Fig 25b and 25d”.

**Fig 26:**
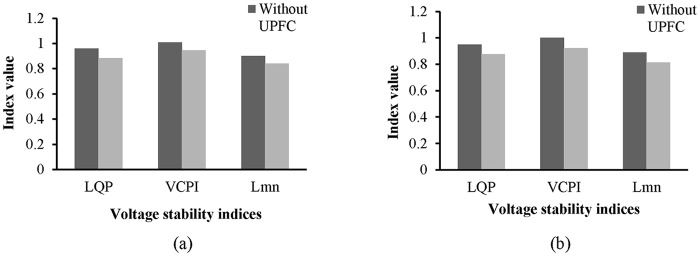
Index values of (a) line 9–8 and (b) line 15–16 of IEEE 39 bus system

The values of the VSIs for lines 9–8 and 15–16 with and without the UPFCs are presented in “Fig 26a and 26b”, respectively. “Fig 26a” reveals that before connecting the UPFCs, when the system is in an unstable condition, and the values of the L_mn_, LQP, and VCPI indices for line 9–8 are 0.893, 0.952, and 1.015, respectively. After the UPFCs are connected, the values of the *L*_*mn*_, *LQP*, and *VCPI* indices for line 9–8 decrease by 0.058, 0.0755, and 0.065, and reached to (0.842), (0.8845), and (0.945) respectively. For line 15–16, “Fig 26b” shows that the values of the L_mn_, LQP, and VCPI indices decrease from 0.874 to 0.801, from 0.938 to 0.8623, and from 1.008 to 0.9215, respectively, in the stable condition, i.e., with the UPFCs connected.

**Fig 27:**
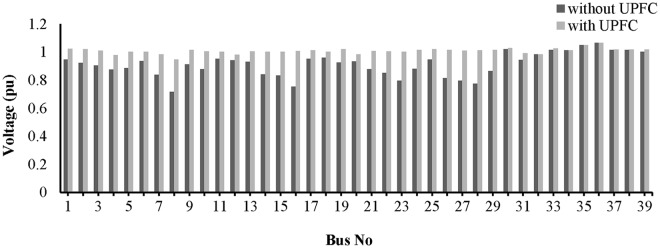
Voltage profile across all the buses of IEEE-39 bus network with UPFCs placement in lines 9–8 and 15–16.

A bar graph is presented in “Fig 27” to show the voltage profiles of the network buses. It can be observed that the voltage profiles of the buses in the network improve with the UPFCs in lines 15–16 and 9–8.

### 3.3 Comparison of static and dynamic methods for selecting UPFC locations

In this section, locations for the UPFCs in the IEEE 14- and 39-bus systems are selected using static methods, and the stability and capacity of the two benchmark systems with UPFCs placed in the locations obtained with the static methods and the proposed dynamic method are analyzed.

#### 3.3.1 IEEE 14-bus system

In the previous section, the location of the UPFC was chosen by examining the VSIs in a dynamic condition, and line 9–14 was selected for a PQ load variation and line 13–14 was selected for a Q load variation. Based on an examination of the VSIs in the static condition, line 6–11 was selected for the location of the UPFC given a PQ load variation and line 1–5 was selected given a Q load variation. These locations are different from those obtained for the dynamic condition. Next, PSO and DE were used to select the locations for the UPFCs using VSIs computed for the static condition and assuming PQ load variations. Using PSO, line 2–4 was selected, and using DE, line 1–2 was selected. These locations differ from those obtained with either of the previous methods. To verify the efficacy of these locations, simulations were conducted both in the steady state and under dynamic conditions with the UPFC connected. In Table 5, the voltage profiles of all of the buses with and without the UPFC are tabulated. From the simulation results, it is clear that the percentage of voltage improvement achieved with the proposed approach (dynamic voltage analysis) is greater than those achieved with either of the static voltage stability analysis methods.

**Table 5:** Voltage profile improvement after placing UPFCs in IEEE—14 bus system by implementing VSIs both in dynamic and static modes.

Bus No	Methods	Dynamic Implementation of VSIs		Static Implementation of VSIs			
	Load variations	PQ load variation	Q load variation	PQ load variation	Q load variation	PSO (PQ load variation)	DE (PQ load variation)
	UPFC locations	Line 9–14	Line 13–14	Line 6–11	Line 1–5	Line 2–4	Line 1–2
	Without UPFC	With UPFC					
	Base voltage (p.u.)	Voltage (p.u.)	Voltage (p.u.)	Voltage (p.u.)	Voltage (p.u.)	Voltage (p.u.)	Voltage (p.u.)
1	1.066	1.065	1.063	1.061	1.06	1.061	1.06
2	1.05	1.049	1.046	1.046	1.04	1.045	1.045
3	1.013	1.012	1.011	1.007	1.008	1.006	1.010
4	1.019	1.021	1.02	1.016	1.015	1.017	1.016
5	1.031	1.03	1.025	1.005	1.018	1.02	1.019
6	1.005	1.004	1.007	0.981	0.9808	0.9907	0.9902
7	1.004	1.003	1.002	0.988	0.987	0.995	0.993
8	1.052	1.042	1.04	1.037	1.038	1.039	1.037
9	1.005	1.004	1.002	0.945	0.963	0.97	0.98
10	1.056	0.9801	0.9886	0.964	0.955	0.963	0.973
11	1.051	0.9805	0.984	0.9615	0.9611	0.9613	0.9701
12	1.01	1.009	1.007	0.9509	0.9506	0.9505	0.9505
13	1.005	1.002	1.01	0.949	0.947	0.946	0.945
14	1.025	0.973	0.9945	0.959	0.956	0.962	0.975

A comparison of the effect of the UPFC on the loadability is presented in Table 6. As shown in Table 6, with the UPFC connected in the various locations, the loadability is greatest when the UPFC is placed at the location obtained with the proposed method. Under normal conditions, the network loadability is 259 MW. When the UPFC is connected across line 9–14, the active loadability improves by 1.08%. The second highest increase is 1.058%, which is achieved with the UPFC placed in line 13–14. For the other UPFC locations, namely lines 6–11, 1–5, 2–4, and 1–2, the improvements in loadability are 1.02%, 1.029%, 1.055%, and 1.012%, respectively, which are less than that achieved with the proposed method.

**Table 6:** Loading capability improvement after placing UPFCs in IEEE-14 bus system by implementing VSIs both in dynamic and static modes.

Method of VSIs Implementation	Types of load variation	UPFC location	Total active power loading capability (MW)		% of improvement
			Without UPFC	With UPFC	
Dynamic Method	Proposed dynamic approach (PQ load)	9–14	259	281.3	1.08
	Proposed dynamic approach (Q load)	13–14	259	274.4	1.058
Static Method	Static approach (PQ load)	6–11	259	264.3	1.02
	Static approach (Q load)	1–5	259	267.1	1.029
	PSO (PQ load)	2–4	259	273.8	1.055
	DE (PQ load)	1–2	259	262.34	1.012

#### 3.3.2 IEEE 39-bus system

With the proposed method (i.e., examining the VSIs for the dynamic condition), the locations identified for the UPFCs in the IEEE 39-bus system were lines 9–8 and 19–16 for PQ load variations and lines 9–8 and 15–16 for Q load variations. However, when the VSIs are computed for the static condition, the resulting locations for the UPFCs are lines 16–17 and 4–5 for PQ load variations and lines 16–17 and 26–27 for Q load variations. The heuristic techniques PSO and DE were used to find the locations of the UPFCs for PQ load variations using VSIs computed for the steady state. With PSO, the locations for the UPFCs were lines 16–17 and 16–24, and with DE, lines 16–17 and 9–8 were selected. The improvement in the bus voltage profiles achieved with the locations for the UPFCs obtained using the proposed dynamic method was greater than those achieved with either of the static methods. The voltage profiles of the buses are tabulated in Table 7.

**Table 7:** Voltage profile improvement after placing UPFCs in IEEE—39 bus system by implementing VSIs both in dynamic and static modes.

Bus No	Methods	Dynamic Implementation of VSIs		Static Implementation of VSIs			
	Load variations	PQ load variation	Q load variation	PQ load variation	Q load variation	PSO (PQ load variation)	DE (PQ load variation)
	UPFC locations	Line 9–8	Line 9–8	Line 4–5	Line 26–27	Line 16–24	Line 9–8
		Line 19–16	Line 15–16	Line 16–17	Line 16–17	Line 16–17	Line 16–17
	Without UPFC	With UPFC					
	Base voltage (p.u.)	Voltage (p.u.)	Voltage (p.u.)	Voltage (p.u.)	Voltage (p.u.)	Voltage (p.u.)	Voltage (p.u.)
1	1.032	1.03	1.023	0.99	1.013	1.015	1.018
2	1.022	1.021	1.02	1.01	1.015	1.018	1.011
3	1.012	1.01	1.01	0.98	0.99	1.002	1.01
4	0.985	0.982	0.9779	0.952	0.957	0.982	0.976
5	1.004	1.001	1.001	0.96	0.98	0.99	0.98
6	1.004	1.001	1.001	0.965	0.975	0.985	0.99
7	0.987	0.983	0.9827	0.95	0.96	0.97	0.988
8	0.989	0.986	0.973	0.945	0.94	0.945	0.968
9	1.0165	1.016	1.014	0.955	0.965	0.975	0.975
10	1.004	1.003	1.003	0.98	0.985	0.99	0.99
11	1.003	1.002	1.002	0.97	0.98	0.988	0.989
12	0.980	0.9793	0.9793	0.945	0.957	0.968	0.972
13	1.006	1.005	1.005	0.97	0.986	0.998	0.998
14	1.002	1.001	1.001	0.998	0.973	0.99	0.99
15	1.0055	1.005	1.001	1.002	0.986	1.001	0.99
16	1.013	1.011	1.008	0.978	1.00	0.987	1.00
17	1.0055	1.005	1.008	0.968	1.00	0.988	0.992
18	1.0015	1.0010	1.001	0.985	0.99	1.00	0.998
19	1.018	1.015	1.02	0.995	1.001	1.005	1.007
20	0.985	0.9829	0.9832	0.958	0.974	0.979	0.979
21	1.015	1.010	1.007	0.947	0.974	0.985	0.975
22	1.004	1.003	1.003	0.968	0.988	1.00	1.00
23	1.002	1.001	1.002	0.976	0.989	0.994	0.99
24	1.02	1.015	1.015	0.988	0.998	1.01	1.011
25	1.025	1.023	1.02	1.001	1.008	1.01	1.013
26	1.028	1.025	1.015	0.978	0.992	1.012	1.009
27	1.018	1.015	1.01	0.976	0.984	1.003	1.001
28	1.023	1.020	1.012	0.998	0.988	0.991	0.997
29	1.018	1.015	1.015	1.01	0.992	0.998	0.98
30	1.028	1.0275	1.0275	1.020	1.0275	1.0270	1.0270
31	0.985	0.981	0.992	0.9522	0.9622	0.9722	0.975
32	0.995	0.9945	0.989	0.9833	0.982	0.9835	0.984
33	1.030	1.029	1.025	1.021	1.024	1.026	1.023
34	1.018	1.015	1.013	1.012	1.011	1.012	1.012
35	1.055	1.05	1.05	1.05	1.052	1.055	1.053
36	1.065	1.06	1.064	1.057	1.059	1.06	1.058
37	1.020	1.016	1.018	1.006	1.009	1.011	1.008
38	1.0175	1.017	1.017	1.005	1.007	1.012	1.004
39	1.023	1.02	1.018	1.011	1.005	1.013	1.01

From Table 8, it can be observed that placing the UPFCs in the locations obtained using the proposed dynamic method results in a greater improvement in loadability than the locations obtained using static methods. Under normal conditions, the loadability of the network is 6084 MW. When the UPFCs are placed across lines 9–8 and 19–16, the active loadability increases by 2.48%. The second highest increase in loadability is 2.25%, which is achieved with the UPFCs connected to lines 9–8 and 15–16. Using the locations obtained with the static methods, the loadability increases by no more than 2.15%, which is less than that achieved with the proposed approach.

**Table 8:** Loading capability improvement after placing UPFCs in IEEE—39 bus system by implementing VSIs both in dynamic and static mode.

Method of VSIs Implementation	Types of load variation	UPFC location	Total active power loading capability (MW)		% of improvement
			Without UPFC	With UPFC	
Dynamic Method	Proposed dynamic approach (PQ load)	9–8	6084	6233	2.48
	Proposed dynamic approach (PQ load)	19–16	6084	6233	2.48
	Proposed dynamic approach (Q load)	9–8	6084	6221	2.25
	Proposed dynamic approach (Q load)	15–16	6084	6221	2.25
Static Method	Static approach (PQ load)	16–17	6084	6208	2.05
	Static approach (PQ load)	4–5	6084	6208	2.05
	Static approach (Q load)	16–17	6084	6202	1.95
	Static approach (Q load)	26–27	6084	6202	1.95
	PSO (PQ load)	16–17	6084	6194	1.81
	PSO (PQ load)	16–24	6084	6194	1.81
	DE (PQ load)	16–17	6084	6217	2.15
	DE (PQ load)	8–9	6084	6217	2.15

## Conclusion

This study investigated an approach for choosing the locations of UPFCs in power system networks based on a dynamic voltage stability analysis. Three VSIs (LQP, VCPI, and L_mn_) were implemented in a PSCAD environment to determine the weakest lines in two example networks, the IEEE 14-bus and 39-bus benchmark networks. To determine the appropriate locations for the UPFCs, two types of load variations, PQ and Q, were used. For the IEEE 14-bus network, line 9–14 was chosen for PQ load variations and line 13–14 was chosen for strictly Q load variations. For the IEEE 39-bus system, two locations were chosen for inserting UPFCs, namely, lines 9–8 and 19–16 for PQ load variations and lines 9–8 and 15–16 for Q load variations. Therefore, it can be concluded that the best locations for the UPFCs depends on the power system conditions.

From the simulation results, it can be observed that placing the UPFCs in the selected locations improved the voltage stability. For example, with PQ load variations the voltage profiles across buses 8, 9, 16, and 19 of the IEEE 39-bus network improved by 28.31%, 15.52%, 29.32%, and 12.24%, respectively, with UPFCs across lines 9–8 and 19–16. Similarly for Q load variations, placing UPFCs in the selected lines improved the voltages across the buses, bringing them closer to their nominal values.

Finally, the locations of the UPFCs obtained using the method proposed in this study resulted in greater improvements in the voltage profiles than the locations obtained with other methods that are based on a static analysis of voltage stability.
